# How a Minimal Learning Agent can Infer the Existence of Unobserved Variables in a Complex Environment

**DOI:** 10.1007/s11023-022-09619-5

**Published:** 2022-12-29

**Authors:** Benjamin Eva, Katja Ried, Thomas Müller, Hans J. Briegel

**Affiliations:** 1 Department of Philosophy, Duke University, 1364 Campus Dr., Durham, NC 27705 USA; 2 Institute for Theoretical Physics, University of Innsbruck, Technikerstraße 21A, Innsbruck, 6020 Austria; 3 Department of Philosophy, University of Konstanz, Fach 17, 78457 Konstanz, Germany

**Keywords:** Concept formation, Theory formation, Projective simulation, Reinforcement learning, Transparent artificial intelligence, Explainable artificial intelligence (XAI)

## Abstract

According to a mainstream position in contemporary cognitive science and philosophy, the use of abstract compositional concepts is amongst the most characteristic indicators of meaningful deliberative thought in an organism or agent. In this article, we show how the ability to develop and utilise abstract conceptual structures can be achieved by a particular kind of learning agent. More specifically, we provide and motivate a concrete operational definition of what it means for these agents to be in possession of abstract concepts, before presenting an explicit example of a minimal architecture that supports this capability. We then proceed to demonstrate how the existence of abstract conceptual structures can be operationally useful in the process of employing previously acquired knowledge in the face of new experiences, thereby vindicating the natural conjecture that the cognitive functions of abstraction and generalisation are closely related.

## Introduction

### Objectives

According to a mainstream position in contemporary cognitive science and philosophy, the use of abstract compositional concepts is amongst the most characteristic indicators of meaningful deliberative thought in an organism or agent (see, e.g., Bermúdez, 2003; Carruthers, 2009; Evans, 1982). Indeed, the verifiable possession of compositional concepts is widely forwarded as a criterion that needs to be satisfied before any substantive doxastic states can be legitimately attributed to non-human animals (see, e.g., Carruthers, 2009; Davidson, 1975; Dreyse, 2011). If one takes this kind of position seriously (as many do), it follows that any system genuinely deserving of the name ‘artificial intelligence’ will possess the ability to effectively traffic in abstract conceptual representations of salient features of its environment. (Indeed, numerous variations of this view have already been articulated and defended in the foundations of AI literature, e.g., Bengio et al. 2013; Lake et al. 2015). In this paper, we address this observation by constructing an explicit example of a simple learning agent that autonomously identifies abstract variables in the process of learning about its environment, before providing a concrete operational semantics that allows external observers to subsequently identify these variables through analysis of the agent’s internal deliberative structures. Moreover, we demonstrate how an agent’s ability to construct and employ these abstract conceptual structures correlates with its ability to employ previously acquired knowledge when dealing with novel experiences.

Beyond the motivation of constructing AI systems that satisfy the criterion of possessing abstract compositional conceptual structures, we take the significance of this work to be threefold. Firstly, by constructing learning agents that are capable of discovering abstract variables in a way that can be objectively identified in subsequent analysis, we take a meaningful step towards developing artificial agents whose reasoning processes are fully transparent, interpretable and communicable. We contrast this with conventional reinforcement learning algorithms, which are by design focused on developing a successful policy—in other words, on learning what to *do*—, and therefore do not typically develop explicitly represented conceptual structures that suggest a straightforward interpretation (see, e.g., Sutton and Barto 1998; Wiering and van Otterlo 2012). By contrast, the agent proposed in the present work structures the information that it gathers in a way that supports an operational interpretative semantics, which is an important first step towards combining the efficient learning abilities of reinforcement learning agents with explicit and communicable symbolic deliberations.

The second point of significance is our observation that agents that have identified abstract variables perform noticeably better at tasks that require them to generalise existing knowledge to deal with new experiences. This both provides a novel operational vindication for the pragmatic and epistemic value of abstract conceptual representations, and solves an existing operational problem regarding the ability of reinforcement learning agents to successfully generalise.

Thirdly, while there already exists a significant literature on identifying latent variables and underlying conceptual structures in the deliberations of e.g. neural networks and deep reinforcement learning systems, this study represents the first attempt to tackle this problem for projective simulation (PS) agents. Given the comparatively simplistic and minimal architecture of PS agents, this is significant insofar as it promises to tell us something about the minimal cognitive machinery that is sufficient for conceptual abstraction in artificial agents.

The paper is structured as follows. In Sect. 2, we describe the kind of learning environment used to investigate the formation and identification of abstract variables. In Sect. 3, we introduce the particular type of reinforcement learning agent to be deployed in those tasks (namely ‘Projective Simulation’ agents) before presenting a novel modification to the architecture of those agents, which provides them with the necessary ‘cognitive space’ for variable identification. In Sect. 4 we formally specify what it means for such an agent to identify variables in the context of the learning tasks described in Sect. 2. In Sect. 5 we present the results of our simulations, which illustrate the efficacy of our variable identification protocol. In particular, Subsect. 5.3 analyses the observed correlation between the existence of identifiable variables in an agent’s deliberations and the ability of that agent to deal with novel experiences in an effective manner. Section 6 provides a conceptual discussion of our results, and Sect. 7 concludes.

## The Learning Environment

### Basic Structure

Our central aims are (i) to enable a learning agent to infer the existence of unobserved variables in a complex environment via dynamic interactions, and (ii) to subsequently develop an operational semantics that allow us to identify a representation of these variables in the agent’s internal deliberation structures. Towards this end, we consider an environment that consists initially of three components:

- A set $\mathcal {S}$ of possible *setups*, i.e., situations on which experiments can be performed. For example, in a context in which the agent is allowed to perform simple classical physical experiments on a range of different objects, each setup $s \in \mathcal {S}$ could represent one object. More generally, each setup *s* represents a different situation that the agent can test via a range of experiments, such that each situation can be distinguished by the results it yields in at least some of the available experiments.
- A set $\mathcal {E}$ of *experiments*, i.e., tests which can be performed on any of the available setups. For example, in the case in which the agent can perform classical physical experiments on objects, one possible experiment could be suspending a given object from a spring and recording by how much the spring is extended.
- A set $\mathcal {P}$ of *predictions* such that each $p \in \mathcal {P}$ corresponds to a prediction of the outcome of exactly one experiment in $\mathcal {E}$. For example, if one of the available experiments is to measure the spin of a particle along the *y*-axis, then $\mathcal {P}$ would contain one prediction corresponding to the ‘spin down along the *y*-xis’ outcome and one prediction corresponding to the ‘spin up along the *y*-axis’ outcome.

A few additional comments regarding the predictions are in order. Since, by assumption, each prediction $p\in \mathcal {P}$ corresponds to exactly one experiment $e\in \mathcal {E}$, one can think of *p* as including a specification of which experiment it pertains to. A prediction *p* is then deemed ‘correct’ for a given setup *s* if, under the experiment *e* for which *p* is a possible prediction, the setup *s* indeed produces the corresponding outcome. Note that, in what follows, we make the simplifying assumption (to be relaxed in future work) that the outcomes of experiments are deterministic, i.e., that each setup/experiment pair predetermines a unique correct prediction. Moreover, we assume that $\mathcal {P}$ contains a complete set of the possible outcomes for every $e \in \mathcal {E}$, in the sense that there can be no combination of a setup $s \in \mathcal {S}$ and an experiment $e \in \mathcal {E}$ performed on it such that the resulting outcome is not among the predictions $\mathcal {P}$. (One can ensure that this holds true even in pathological cases, such as an attempt to measure the spin of a particle in the eventuality that no particle is present, by formally including one prediction to the effect of ‘not applicable’.) Finally, we note that, throughout the present work, we consider the spaces of experiments and predictions to be discrete. Scenarios with continuous parameters can be made compatible with this framework by suitable binning. The possibilities for handling continuous input and output spaces in the specific learning framework that we will use are explored in Jerbi et al. (2021) and Melnikov et al. (2018).

Once the environment has been fully specified via a choice of $\mathcal {S}, \mathcal {E}$ and $\mathcal {P}$, agents interact with it in the following way. Each round of interaction begins with the agent being presented with a single setup $s \in \mathcal {S}$ that is drawn from a fixed probability distribution over $\mathcal {S}$, which we assume to be uniform.^1^ Upon being presented with *s*, the agent is asked to make a prediction $p \in \mathcal {P}$ (which, as detailed above, implicitly includes a choice of an experiment *e*). Finally, the agent receives a reward if and only if their prediction is correct for the setup. For example, the agent could be presented with a particular object *s* and asked to make a prediction for any one of the available experiments that could be performed on that setup, e.g., holding it next to a magnet. They would then be rewarded if and only if their prediction matched the outcome of that experiment, e.g., being attracted by the magnet.

The above learning environment is reminiscent of classic reinforcement learning tasks, in which success is equated with efficiently learning how to choose the correct option (prediction) for all possible inputs (setups), i.e., with efficiently learning how to maximise rewards. However, in our approach, this is only the first step of what constitutes successful learning. Rather than merely learning how to make correct predictions, our central success criterion is that the agent develop transparent and easily interpretable conceptual representations of those aspects of their environment that play a role in determining the outcomes of experiments.

To make this success criterion precise, we will introduce one additional component in our description of the environment. It is based on the observation that each setup could be uniquely identified by a specification of the values of a number of suitable abstract variables, e.g., the size, shape and composition of an object. Crucially, we do not assume that the agent can perceive the values of these variables, or even that they are aware of the fact that a description of the observed setup can be compressed in such a way. Our goal is precisely to construct an agent that can infer the existence of such variables even if the setups are presented as mere atomic labels that carry no intrinsic meaning. Formally, we assume that, in addition to the three components $\mathcal {S, E, P}$ specified above, the environment also contains a ‘hidden’ fourth component, namely

- A set $\mathcal {V}$ of ‘hidden’ (or latent) variables, i.e., variables which are never explicitly presented to the agent, but which are sufficient to determine the outcomes of all experiments.^2^ Each setup $s \in S$ can be equated with a vector specifying exactly one value for each of the variables in $\mathcal {V}$, and each experiment is assumed to test one and only one of the variables in $\mathcal {V}$, although there may be multiple experiments testing the same variable. For instance, if there are two variables with two values each, then there will be four setups corresponding to the four possible configurations of the values of the variables in $\mathcal {V}$, i.e $s_{1} = 00, s_{2} = 01, s_{3} = 10, s_{4} = 11$. There will also be at least two experiments, each corresponding to one variable, where the outcome of each experiment is determined by the value that the given setup entails for the corresponding variable.

The problem of unobserved variables is also relevant to the field of machine learning (specifically reinforcement learning), in the context of partially observable Markov decision processes (POMDPs, see, e.g., Poupart, 2012). In such processes, the input available to the agent does not contain sufficient information to completely characterise the state of the environment, or to make deterministic assessments of the consequences of possible actions. By contrast, in the scenario considered here, the input (the setup *s*) does completely specify the state, in the sense that *s* determines with certainty the outcomes of all possible experiments that could be performed on it. What the agent is searching for, however, is auxiliary variables that help *structure* the relations between the various setups in $\mathcal {S}$ and the corresponding predictions.

### Concrete Scenario Used in Training Agents

**Fig. 1:**
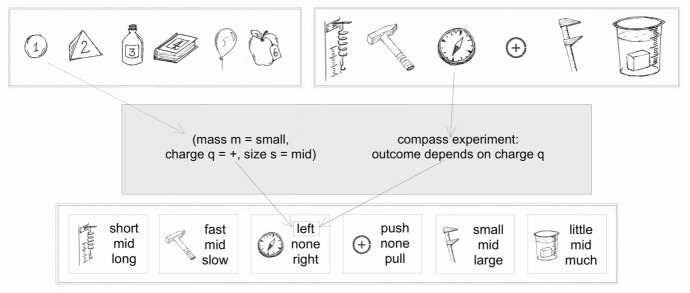
A task environment with hidden variables and rules to be discovered: the agent receives objects (top left, numerical labels 1 through 6) on which it can perform a range of experiments (top right), whose outcomes (bottom) it attempts to predict. What is hidden from the agent (grey box) is that each object can be described by a vector of values, namely its mass, charge and size, and each experiment can be predicted given the value of one of these variables: when suspending the object from a spring (‘scale experiment’) or hitting it to impart a given momentum (‘momentum experiment’), the outcome depends on the object’s mass; when passing it near a compass or placing it near a test charge, the results depend on the object’s charge, while the outcomes of grasping the object or submerging it in a bucket of water depend on its size

To illustrate these ideas, we now provide a concrete example of a learning environment containing hidden variables. This scenario will also be used as the default case in our subsequent analysis of the agents’ learning capabilities. It is illustrated visually in Fig. 1.

In our default scenario, each setup consists of an object that the agent can experiment on, which is characterised by $|\mathcal {V}| = 3$ hidden variables: mass, size and electric charge. (Since objects can in principle have different densities, mass and size need not be correlated.) Each of these properties predicts the outcomes of two different experiments: for example, electric charge predicts what force the object will experience when placed next to a given test charge, and also how much the object will deflect a compass needle when moving past it at a given speed and distance. Mass predicts what will happen if an object is hit, imparting a fixed amount of momentum, or if it is placed on a scale in a fixed gravitational field, and similarly an object’s size predicts its behaviour in two other experiments. Overall, this environment admits $|\mathcal {E}| = 6$ experiments that the agent can perform. We assume that the possible values of each variable are coarse-grained into three distinct values; for example, the variable ‘electric charge’ can take the (coarse-grained) values ‘positive’, ‘negative’ and ‘neutral’ and the variable ‘size’ can take the values ‘big’, ‘small’ and ‘medium’. These values are reflected in corresponding outcomes for each experiment, so that the number of predictions corresponding to each experiment is also^3^ 3. This gives rise to a total of $|\mathcal {P}| = 6\cdot 3 = 18$ predictions that the agent can choose from, of which 6 will be correct for any given object. The expected success rate for random guesses in this environment is therefore 1/3. Since setups (objects) are identified with configurations of the values of the variables in $\mathcal {V}$, it follows that $|\mathcal {S}| = 27$, i.e., there are 27 distinct objects on which the agent is able to experiment, and each of those objects instantiates one of the 27 possible configurations of the values of the ‘charge’, ‘size’ and ‘mass’ variables.

The agent’s interaction with this environment proceeds as outlined above: the agent is presented with a randomly chosen object, which is labelled simply with an integer between 1 and 27; the agent then chooses an experiment, makes a corresponding prediction and finally receives a reward if that prediction was correct for the given object. This process is iterated long enough for the agent to eventually encounter all the available objects and learn about them. Specifically, the agents whose results are analysed in the following are given $T=5*10^6$ rounds of interaction with the environment in order to learn, in the default case.

In the standard reinforcement learning paradigm, the criterion for success in the kind of learning task described here would be that the agent successfully learns how to make the correct predictions for all object/experiment pairs. This is a purely operational criterion that can be straightforwardly accomplished in a reinforcement learning setting by implementing a learning dynamics that increases an agent’s disposition to make particular predictions in proportion to the extent to which those predictions have been rewarded in the past (and implementing some form of greed avoidance). Indeed, as we will see later, this first goal can be accomplished relatively easily. However, we have also introduced a second criterion for success, which is the central aim of the present work: that the agent, beyond learning how to reliably predict the outcomes for all object/experiment pairs, also comes to identify that there are three hidden variables that determine which predictions will be correct for each object/experiment pair.

### The Value of Understanding

With this formal description of the environment in hand, it is worth pausing to reiterate a few of the central motivations behind this second success criterion. Firstly, identifying the variables at play in the agent’s deliberation is a crucial first step towards rendering the agent’s deliberations genuinely *transparent*, *interpretable* and *communicable*. Secondly, there is a significant difference between an agent that merely memorises which predictions were rewarded for which object/experiment pairs and an agent that has identified that there exist significant unobserved variables—which we might identify as ‘mass’, ‘size’ and ‘charge’—and makes predictions on the basis of which value a given variable takes for a given object. (For example, the agent predicts that the second object will present a reading of ‘high’ in the scale experiment because it already knows that (i) there exists a variable that predicts the outcome of the scale experiment (‘mass’) and (ii) that, based on the momentum experiment, the object has high mass.) It is natural to say that the second agent possesses a genuine *understanding* of its environment, in a sense which is absent for the first agent.^4^ A similar sentiment is expressed by Bengio et al. (2013), who write

An AI must fundamentally understand the world around us, and we argue that this can only be achieved if it can learn to identify and disentangle the underlying explanatory factors hidden in the observed milieu of low-level sensory data (Bengio et al. 2013, 1798).

Thirdly, many cognitive abilities are grounded in the ability to describe one’s environment in terms of abstract conceptual representations. Saliently, the ability to generalise previously acquired knowledge to deal with new experiences seems to be intimately connected to the ability to represent significant properties of one’s environment in terms of abstract variables. (This intuitive conjecture is empirically vindicated in Sect. 5.3.) More generally, there are numerous pragmatic and theoretical motivations for regarding the identification of abstract variables corresponding to the environment’s hidden variables as a success criterion for explorative learning agents. In Sect. 4, we will provide a concrete formalisation of this second success criterion for a particular kind of reinforcement learning agent. First, we turn to specifying the precise cognitive architecture of those agents.

## The Learning Agent

We will work within the context of the projective simulation (PS) framework for artificial intelligence agents, which was first proposed by Briegel and De las Cuevas (2012). This framework aims to provide a concrete example of what it means to be a deliberating agent: entities that can *act* on their environment, thereby generally changing its state, and, more importantly, that make their *own decisions* in the sense that they are not pre-programmed to take particular actions under given circumstances, but instead are flexible and develop their own action and response patterns.

While one of the achievements of the PS framework is to provide a concrete, explicit model of agency, the agents’ ability to learn has been a point of considerable interest, having been tested against more utilitarian reinforcement learning algorithms on a number of benchmark problems (see, e.g., Briegel & De las Cuevas, 2012; Mautner et al., 2015; Melnikov et al., 2018). The broad conceptual-mathematical basis also supports much more diverse applications, ranging from the autonomous development of complex skills in robotics (Hangl et al., 2016, 2020) through modelling of collective behaviour in animal swarms (López-Incera et al., 2021; Ried et al., 2019) to the modelling of the formation of stimulus equivalence classes in humans (Mofrad et al., 2020). The PS model has also been applied to the control of quantum systems (Nautrup et al., 2019; Tiersch et al., 2015; Wallnöfer et al., 2020) and the design of new experiments (Melnikov et al., 2018).

The interaction of the agent with its environment is formalised following the general framework of reinforcement learning (RL; see, e.g., Sutton & Barto 1998): the learner receives a *percept* that encodes some information about the state of its environment, based on which it chooses an *action*, and, if the action puts the environment in a state that satisfies some pre-defined success criterion, the learner is given a *reward*. A classic example of reinforcement learning is the grid world task, wherein the agent must navigate a maze: at each time-step, it perceives its current position, chooses to take a step in some direction, and, if this brings it to a goal that is located somewhere in the maze, receives a reward. This pattern of interactions fits in naturally with the structured learning environment outlined in Sect. 2, with percepts specifying the setup and actions being the choice of a prediction. Only a small modification is required regarding rewards: if an agent is supposed to discover patterns and hidden variables by making predictions about the world, it should not rely on rewards provided by the environment, but instead be endowed with an internal mechanism by which it essentially rewards itself if the prediction was correct. (The idea of a learning process that does not primarily aim to achieve an externally supplied reward, but instead encourages a learner to explore its environment simply for the sake of obtaining more information (although that may turn out to be useful for reaching more external rewards in the future), was incorporated from developmental psychology into reinforcement learning under the term *intrinsically motivated learning* (Barto, 2013; Oudeyer et al., 2007). More specifically, intrisically motivated learning in RL often refers to mechanisms that guide the learner towards situations that maximise the gain of new information, which one might describe as curiosity. For the purpose of the present work, however, it is sufficient to consider an agent that simply rewards itself whenever it makes a correct prediction.)

**Fig. 2:**
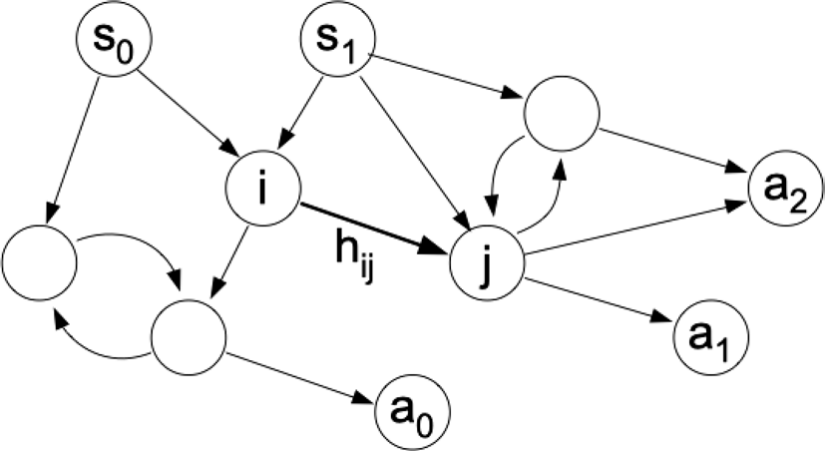
A PS agent’s memory of its interaction with the environment is summarised in the *episodic and compositional memory* (ECM): a network of *clips* (the network depicted here contains 10 clips), including in particular percept clips (denoted $s_i$) and action clips ($a_i$). This network also includes additional ‘intermediate clips’ (of which the clips labelled *i* and *j* are two examples) that lie in between the percept and action clips in the network. Deliberation is realised as a random walk over clip space, starting at a percept and terminating at an action, with the probabilities of hopping from clip *i* to clip *j* governed by the weights $h_{ij}$ of the relevant edges. If a reward is received, the edges traversed to reach that decision are strengthened

Let us now turn to the internal mechanism by which agents decide on an action given a percept, which is the defining feature for which projective simulation is named: PS agents simulate (or project) conceivable developments that, based on past experience, could arise from the present percept. Their simulation favours those sequences that have been rewarded in the past, so as to arrive at an action that is also likely to carry a reward. In order to ensure the autonomy and flexibility of the agent, the simulation is not based on some predefined representation of the environment, but instead on episodic ‘snippets’ —termed *clips*—from the agent’s own experience, which could represent percepts, actions or combinations thereof. The deliberation process consists of a random walk over clip space, starting at the clip that represents the percept currently being presented and terminating when an action clip is reached and the corresponding action realised. A generic example of such a clip network is illustrated in Fig. 2.

In order to adapt its responses to an environment—that is, to learn—the agent must be able to modify how the random walk over clip space proceeds. To this end, each edge from clip *i* to clip *j* is given a (positive, real-valued) weight, termed the *hopping value* or *h-value* for short and denoted $h_{ij}$. These weights govern the probabilities with which the walk proceeds from clip *i* to clip *j*:

1 $$\begin{aligned} P(j|i) = \frac{h_{ij}}{\sum _k h_{ik}}, \end{aligned}$$

with all weights set initially to $h_{ij}=1$. PS agents learn primarily by modifying the weights of edges: if a deliberation process going from percept *s* to action *a* leads to a reward *R*, then all the edges traversed as part of this deliberation are strengthened, i.e., their *h*-values are increased. In general, this is balanced by *forgetting*, which decreases the weights of all edges by a factor $1-\gamma$, driving them back to their initial weight of $h_{ij}=1$, so as to gradually eliminate unused connections. Combining these two mechanisms, the update rule for *h*-values reads

2 $$\begin{aligned} h_{ij}^{\left( t+1\right) }-1=\left( 1-\gamma \right) \left( h_{ij}^{\left( t\right) }-1\right) +{\left\{ \begin{array}{ll} R^{\left( t\right) } &{} \text {if used,}\\ 0 &{} \text {if unused,} \end{array}\right. } \end{aligned}$$

where $R^{\left( t\right) }$ denotes the reward received at turn *t*.

The network of connected clips inside a PS agent is termed *episodic and compositional memory* (ECM), based on two noteworthy properties: firstly, the sequence of clips that are excited during a random walk can be understood as a simulation of an ordered sequence of events, or an *episode*. Secondly, the set of clips over which the walk proceeds is not static, but can be augmented by creating new clips, either by *composing* existing ones or by adding entirely new clips that can be used to represent novel content. This second possibility, of additional clips that represent neither percepts nor actions, will enable our agents to form novel concepts. (While such clips can in principle be created dynamically, during the learning process, the present work focuses on how existing clips can come to represent an agent’s knowledge of hidden variables, leaving the exploration of clip creation to future work.)

### Enabling Learning Agents to Handle More Complex Environments: Connections to Existing Work

**Fig. 3:**
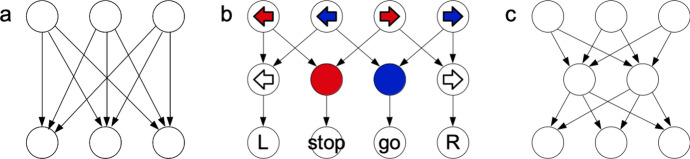
Various possible ECM structures connecting percept clips (top layer), action clips (bottom layer) and (for **b** and **c**) intermediate clips. The two-layer network in **a** is the simplest possible architecture, and contains no intermediate clips. The network in **b** contains intermediate ‘wildcard clips’, which represent information about a subset of components of the percept (described below). **c** depicts the three-layer architecture proposed here, with a layer of intermediate clips that have no a-priori initial meaning, but will come to represent values of hidden variables

In the simplest agents, the ECM has just two layers, representing percepts and actions, with connections proceeding simply from percepts to actions, and their strengths encoding which is the preferred response to each input. Such a structure is shown in Fig. 3a. However, more complex tasks can generally be solved better with more sophisticated structures. By way of illustration, this section summarises a simple learning task that was previously posed to PS, and that resembles the abstraction task of the present work, before discussing previously proposed modifications that enable PS to handle this challenge.

The task of interest is the infinite colour game, introduced by Melnikov et al. (2017). In this environment, the agent is shown a two-component percept, featuring an arrow that points in a certain direction (left or right) and is painted in one of (countably) infinitely many colours. The agent then has the choice of moving left or right (ostensibly to defend one of two doors against an attacker) and is rewarded if it chose the correct action. The ‘hidden structure’ in this environment is that the correct choice is telegraphed solely by the direction of the arrow, whereas the colour information is irrelevant to the task. The challenge for the agent is to learn to disregard colour, which would allow it to achieve perfect success in its responses even if it has never encountered a particular percept (that is, that combination of direction and colour) before.

To solve this problem, Melnikov et al. (2017) introduced an architecture where the agent dynamically generates *wildcard clips*: additional clips that are added to the ECM between the layers of percept and action clips, representing either only a direction without specifying a colour or only a colour without specifying a direction (or, most generally, neither a colour nor a direction, i.e., a completely uninformative clip). The structure is illustrated in Fig. 3b. Notably, the wildcard clips are connected to the two-component percept clips according to a fixed rule, namely connecting only to those percepts that contain the direction (resp. colour) in question. In order for such an agent to be successful in a given environment, the environment must have two key properties: the percept space must be formed by products of several components (or categories), which the agent must be able to perceive as independent pieces of information, and the reward rule must be such that disregarding a subset of these components is a useful strategy for determining the correct actions. By capitalising on these properties, wildcard PS performed significantly better than chance on the infinite colour game, which is not possible for standard (tabular) reinforcement learning (including basic PS), since the agent is presented with an entirely new percept at each time step. Here we want to abandon the assumption that the structure of the relevant variables is known a priori and aim instead to construct an agent that is able to *infer* the structure of the variables from its interactions with the environment.

Considering the above properties of the PS framework, we note that, while the architecture of PS agents supports reinforcement learning (RL), it differs from conventional, more widely used algorithms for RL or for machine learning in general in several important ways. Contrasting with RL algorithms such as SARSA or Q-Learning (Sutton & Barto, 1998), which essentially tabulate the expected rewards for each percept-action pair, the additional internal structure of PS agents supports a more complex understanding of the environment. (In the case of environments that do not require such complexity, such as the classic benchmark tasks ‘grid world’ and ‘mountain car’, PS also achieves results that are at least comparable to conventional, tabular RL algorithms (Melnikov et al., 2018).

Another important and influential framework for machine learning employs artificial neural networks (NNs). These networks also possess several layers or more convoluted structures and may, on first view, look quite similar to the ECM in Fig. 3c. However, the two structures function along very different lines. For one, training NNs (by backpropagation) requires the learner to know what the outcome should have been (i.e., a supervised learning setting), whereas PS can learn by trial and error, i.e., being informed only whether it made the correct choice. With respect to the internal functioning, a single deliberative process in an NN excites many neurons, often at the same time, with information being encoded in the *pattern* of excitations, whereas individual neurons typically carry no clear meaning. By contrast, in an ECM, exactly one clip is excited at a time, and any single clip can carry all the information involved in the deliberative process at that time (for example, the entire percept or a complete specification of the action that the agent is deciding to take). This difference in functioning and semantics becomes especially relevant when one is concerned with interpretability: due to the delocalised way in which a NN represents information, it takes considerable effort to trace or explain how it reached the conclusion it did (Alvarez-Melis & Jaakkola, 2018; Biran & Cotton, 2017; Molnar, 2022; Samek et al., 2017; Sellam et al., 2019). PS, on the other hand, clearly reveals what path the deliberation took, passing through particular intermediate clips that can—as we will see in the following—be endowed with an objective interpretation in terms of hidden variables.

### Specific Architecture of Our Agents

As we stressed in the previous section, the ambition of the present work is not simply that the agents learn to make correct predictions, but rather that they develop some internal representation of the hidden variables that underlie such predictions. At first blush, there is no obvious way to encode such representations in the simplest two-layer structure that is characteristic of the ECMs of basic PS agents. So, in order to support such representations, we propose an agent whose ECM consists of *three* layers: an initial layer of percept clips (with one clip to represent each possible setup; in the default scenario, 27), a final layer of action clips (representing the predictions for the various experiments, by default 18), and, between them, a layer of *intermediate clips* (denoted $\mathcal {I}$).

We assume that these three layers are connected in a particular way, as illustrated in Fig. 3c. (Note that the following specifies only which connections *exist* in the ECM. The weights of the connections, on the other hand, which effectively guide the agent’s choices and which will serve as a basis for identifying hidden variables, develop during the learning process.) In our agents, each clip in one layer is connected to all the clips in the layer(s) immediately before and after, but not to any clips in the same layer or in more distant ones. Moreover, all connections are directed from percepts towards actions, so that the ECM is acyclic. Thus, every path from a percept clip to an action clip passes through exactly one intermediate clip on the way, and for every percept-action pair there is one path through each intermediate clip. Note that, while the ECM depicted in Fig. 2 does contain intermediate clips, it does not have the same kind of layer structure as our agents, since some of these intermediate clips are connected to one another and hence that random walks through this ECM can include cycles.

Regarding the number of intermediate clips, we require only that it be no greater than the number of possible setups (percepts) the agent may encounter, and otherwise leave the number of clips in $\mathcal {I}$ unconstrained. This requirement is related to the natural interpretation of the intermediate clips. Intuitively, the idea is that each intermediate clip denotes a possible *label* for a given situation (percept/setup). When the agent encounters a setup $s \in S$, they first have to choose a ‘label’ for that experience. This is formalised as the random walk through the agent’s ECM transitioning from *s* to some $i \in \mathcal {I}$. Based on the label *i*, the agent then chooses an action—formally, by transitioning from the intermediate clip *i* to an action clip *a*.^5^ Note that such labels may well be shared by various setups, but each meaningful label must be attached to at least one setup. For this reason, there is no point having more labels than there are setups to assign them to; hence the requirement that $|\mathcal {S}| \ge |\mathcal {I}|$. In the present work, we consider agents whose number of intermediate clips is equal to the number of hidden variables times the number of values that each variable can take. Preliminary tests suggest that having fewer intermediate clips than that is a significant obstacle to abstraction, whereas a larger number of clips leads to a slight reduction in learning efficiency, but does not pose any fundamental problems. We intend to explore this in more detail in future work.

An additional feature of our proposed agents is a ‘boredom’ mechanism for greed avoidance, which addresses the following problem: once an agent has made the connection from a particular setup *s* to one prediction *p*, which pertains to a particular experiment *e*, the most effective way for the agent to continue reaping rewards is to simply repeat prediction *p* every time it encounters setup *s*. However, we want the agent to explore what would be the correct predictions for other experiments $e'$ as well. (The dilemma of balancing between these two goals is well-known in machine learning, where it is usually termed the ‘exploration vs exploitation’ tradeoff.) To favour exploration, the agent is endowed with ‘boredom’: if, for a give setup *s* and a particular experiment *e*, the agent has come to favour one of the predictions that pertain to *e* over the others with high probability, then experiment *e* is deemed boring with this setup. Formally, for a given *s*, any prediction that pertains to an experiment that is deemed ‘boring’ is rejected, with the deliberation process simply being reset until it produces a prediction about a non-boring experiment. (Once all experiments have reached ‘boring’ status, this mechanism ceases to apply.) We note that this rejection and resetting is an internal process applied by the agent itself. As far as the environment is concerned, the agent eventually produces a single prediction, which is guaranteed to pertain to an experiment that is not boring.

In order to highlight the capabilities that this architecture affords, we will compare the *three-layer agents* described so far against simpler *two-layer agents*, which lack an intermediate layer (see Fig. 3a). We will show that three-layer agents develop patterns of connection weights that can be interpreted as representing the environment’s hidden variables and perform significantly better than chance on generalisation tests, whereas their two-layer counterparts are incapable of either of these feats.

### Comparison to Related Work

In this subsection, we briefly compare the approach developed here to some relevant work from the extant literature. Before addressing how our work fits into the context of existing work on neural networks (NNs) more broadly, we begin by discussing one particular recent paper that tackles a question closely related to our own: Iten et al. (2020) trained NNs in such a way that they managed to ‘extract simple physical concepts from experimental data’. An obvious difference between Iten et al. (2020) and the present work is the implementation that supports the learning process (artificial neural networks in one case, projective simulation in the other). However, a more interesting point for the present discussion are the conceptual differences regarding *what* is learned in each case, rather than how. One fundamental difference is that we consider agents that *explore* their environment by interacting with it and, accordingly, adopt the paradigm of reinforcement learning. By contrast, continuing with the example of damped oscillators, Iten et al. consider an algorithm that is fed pre-recorded data—one might imagine being given a notebook with observations made in a laboratory, but no opportunity to go to the lab and experiment oneself. While learning from pre-recorded data is a powerful paradigm that has achieved great success for certain classes of problems, it requires the implicit assumption that there was already some entity that gathered the data, and, more fundamentally, that *identified relevant variables* whose values should be recorded for subsequent analysis. The recent paper by Chen et al. (2022) shows that it is feasible for a broad range of systems to infer, first, a system’s intrinsic dimensionality and, second, a predictively useful set of state variables, from video recordings of the system’s behaviour. This avoids the step of having to assume a known basis of relevant variables by taking the video recordings as a kind of *lingua franca* for the study of dynamical systems. The results reported in this study are impressive and point the way to fruitful applications in may areas. Still, the choice of video as *lingua franca* also embodies a—subtle—dependence on pre-selected variables, and the data-heavy approach depends on the availability of previously gathered recordings rather than on an agent’s direct interaction with a given environment.

It is this pre-requisite for data-based learning that our agents address: they start from a setting where it is not known how a stream of complex sensory input should be decomposed into independent, meaningful variables. This problem is not as far-fetched as one might think: in the early development of various theories, for example quantum mechanics and electromagnetism, it was a point of considerable debate which variables or concepts might be useful in talking about the subject, and progress was only made by experimentation—that is, by interacting with the systems under study. In our formal framework, this absence of pre-existing variables is reflected in the fact that we consider percepts as being labelled by unique, atomic indices rather than vectors consisting of well-defined components. Our agents take the first basic step of classifying these percepts by ascribing to them operationally meaningful labels, which, crucially, have a particular structure, with groups of labels forming a mutually exclusive and jointly exhaustive classification of percepts. We argue that this property of a set of labels is the defining feature that allows one to interpret them as representing values of some unobserved variable. In this sense, our agents can discover the existence of hidden environmental variables.

The question of how one might infer the values of such variables from the available perceptual data is a second, distinct step in learning about the environment. Our agents, facing an environment that is less challenging in this regard, can essentially memorise the value of each variable for each percept. Iten et al. offer a more sophisticated approach to this part of the problem, implicitly modelling the relation between the new-found variables by learning to compress families of curves relating their values. However, we note that such compression can only be successful if one ensures that all curves are drawn from the same family (for example, recording the position over time for damped oscillators). In order to ensure that each data-set instantiates the relation between the same pair (or set) of variables and that other relevant circumstances are kept constant throughout, one must once again first identify the relevant variables for the system under study. It is the ability to perform this first, more fundamental step, of autonomously discovering that unstructured, atomic percepts admit a decomposition into meaningful variables, that is missing in the aforementioned examples using neural networks.

Going beyond the work of Iten et al. in particular, the challenge of enabling interactive agents to discover patterns and latent variables has also been tackled in a broader body of work centered around the possibility of enhancing RL agents with neural networks. In *Deep RL* (Mnih et al., 2015), deep NNs are used as function approximators to compress the action-value-function (or its equivalent) learned by an RL agent. This approach then allows one to discover latent variables in an RL-type setting by using any of the existing methods from unsupervised learning.

For example, the cascade correlation neural network learning architecture (Fahlman & Lebiere, 1990) offers a way to build a NN whose architecture is adapted to the particular problem being learned, with individual neurons (bits in the circuit implementation) being trained in such a way that they come to capture remaining free parameters in the data distribution. Despite its extremely simple and transparent implementation, this approach performed significantly better than its contemporaries on the two-spiral problem (due to Wieland; as cited in Fahlman & Lebiere, 1990), which, due to its highly non-linear nature, highlights precisely the challenge of introducing useful hidden units.

More recent efforts towards discovering meaningful structures in NNs focus on learning *disentangled representations*: under the assumption that the model generating the data (the environment) contains a set of latent variables that are statistically independent of each other, the goal is to formulate learning rules such that these so-called *factors of variation* come to be represented by independent parameters in memory as well (Bengio et al., 2013). This is not only conceptually appealing, but also, at a more practical level, can sometimes lead to improvements in performance (van Steenkiste et al., 2019). However, this approach is not above criticism: Locatello et al. (2018) show that ‘the unsupervised learning of disentangled representations is fundamentally impossible without inductive biases on both the models and the data’ and further present experimental data suggesting that disentanglement does not, in fact, decrease the sample complexity of learning for downstream tasks.

A prominent approach for achieving the goal of disentangled representations in an unsupervised setting are specific Variational Auto-Encoders (Higgins et al., 2017a) ($\beta$-VAEs), which combine the conventional goal of autoencoders—to compress inputs to a latent representation that allows accurate reconstruction—with additional constraints on the capacity of the latent representation and a cost function promoting *independence* between the latent degrees of freedom. In an unsupervised setting, this leads to ’state-of-the-art disentanglement performance compared to various baselines on a variety of complex datasets’ (Higgins et al., 2017a). The benefits of disentangled representations can then be reaped in a RL setting by combining $\beta$-VAEs with a conventional RL architecture, as detailed, e.g., in Higgins et al. (2017b).

Comparing the present work to the above approaches, we note firstly that the problem statements are somewhat different: while deep RL in general supports any task that can be cast in the percept-action-reward framework, the present work assumes a rather more rigid structure of experiments and predictions, with each experiment revealing the value of one latent variable. However, we note that this particular subclass of RL tasks was designed specifically as a toy problem for the purpose of studying how well an agent can identify latent variables, and as such may allow one to formulate a clearer account of an agent’s performance than if one used more general tasks. For example, the number of latent variables and the number of values each variable can take, which are arguably important degrees of freedom in specifying a latent variable discovery problem, are explicit, independent parameters in our task environment. In a similar vein, our task environment allows one to capture the possibility that, in an environment with several latent degrees of freedom, a particular *partition* may be more natural than others, based on the fact that different degrees of freedom are revealed in different experiments. For example, while the trajectories of two free particles can technically be parameterised by any linear combination of their initial positions and momenta, it is a valuable additional insight that the most helpful way of partitioning this information is by separating into independent parameters for each particle. If the learning task provides no clues about the correct partition—and one cannot generally assume that they do, by default—, then it is not surprising that agents would struggle with identifying the most appropriate partition (see e.g. Iten et al., 2020).

Turning to the architecture used to solve the task, we note that our architecture is more minimalistic in the sense that it requires a smaller network of one-shot nodes and that it functions according to rules that require only very basic computational primitives, which could even be realized by leveraging simple physical processes in an embodied implementation (see, e.g. Flamini et al., 2020). This is appealing, for one, because it raises the possibility of reducing the computational cost, which tends to be a dominant limiting factor in current work with machine learning in general. Moreover, formulating an architecture that requires only basic computational primitives is appropriate if one’s goal is to explore (and exploit) possible parallels between machine learning and biological neural processes. Most importantly, however, exploring a second, alternative way of solving the same problem—of discovering latent variables in a RL setting—can provide valuable clues as to which elements of the architecture are actually necessary in order to make disentanglement work. We feel that this is a particularly valuable contribution that the present work can offer.

One of the most influential attempts to automate the generation of novel scientific concepts, theories and laws is due to Langley et al. (1987), who developed a series of algorithms known as the ‘BACON Systems’ (after Francis Bacon). Langley et al. set themselves the ambitious project of developing a general normative theory of scientific discovery that formally delineates the rational mechanisms by which scientists generate novel hypotheses in the face of experimental evidence. In order to achieve this goal, they identified a number of simple heuristics that play a salient role in the generation of prominent discoveries from the history of science. They subsequently combined these heuristics to develop a series of general function finding algorithms (the BACON systems) that automatically identify functional relationships between the given features in the relevant body of data. Some of these heuristics specify when new complex variables should be considered, based on the relationship between the features that have already been considered. For example, one heuristic stipulates that, when the value of one feature increases as the value of another feature decreases, the product of those two features should be considered. Another stipulates that when the value of one feature increases monotonically with the value of another feature, the quotient of those two features should be considered. Langley et al. apply the BACON systems to reproductions of the data that was available to various physicists prior to their formulation of famous laws and hypotheses, and show that BACON is able to reproduce the relevant discoveries. By way of illustration, when BACON is presented with a table of data representing the distance to the sun (*D*) and period (*P*) of the planets in our solar system, it uses three simple heuristics (including the two listed above) to eventually derive Kepler’s third law, which states that $\frac{D^{3}}{P^{2}} = k$ (where *k* is a constant).

At first glance, one might think that BACON is achieving something similar to the system described in this article. Specifically, BACON is nominally able to autonomously identify variables, such as $D^{3}$ and $P^{2}$, that are crucial for systematising the existing data, but were not explicitly presented to the system (the initial data table contained only two features: *D* and *P*). Thus, it may seem that BACON preempts our stated goal of automating the process of identifying relevant scientific variables. But there are a number of major conceptual and technical distinctions between the PS system implemented here and the BACON algorithms.

Firstly, even in the more sophisticated later versions of the BACON algorithms, the variables that the system is capable of identifying are always functions of the features that were presented in the initial data. So the BACON systems always rely on an initial compression of the environment into relevant features, and subsequently generate new variables/hypotheses/laws, *on the basis of that representation*. In contrast, the PS system described here does not rely on or have access to any comparable a-priori representation of its environment in terms of features. The variables that it identifies are therefore genuinely *new* in a sense that is not true of those identified by the BACON systems. They are not simply functions of variables that have already been externally given to the system. Rather, they represent the system’s best attempts to compress what it has learned about its environment in a communicable and transparent way. Secondly, the PS system presented here also works in a fundamentally different way to the BACON system, insofar as BACON is concerned with systematising an explicitly presented body of observational data, while the PS system never addresses a clearly defined body of observational data, but rather simply undergoes iterated interactions with its environment, and must identify variables that allow it to summarise what it has learned through those interactions. Thirdly, the heuristics employed by the BACON system are explicitly designed for discovering functional relationships between the features that comprise the data. The PS system described here does not rely on the supposition that the relevant latent variables stand in functional relationships to one another. In future work, we show how this system is capable of identifying functional and probabilistic relations between the variables that it identifies.

Overall then, it is clear that although the PS variable identification system described here overlaps with existing machine learning paradigms in terms of both its methods and its objectives, it also aims to solve problems that are not yet fully addressed by any of those paradigms. We turn now to further discussing the extent to which the PS system itself can truly be said to have solved those problems.

## Variable Identification

In the standard environment described in Sect. 2.2, the agent is presented with an integer index specifying one of 27 possible setups, before subsequently choosing one of 18 available predictions (each of which pertains to one of 6 available experiments). The random walk leading to that decision consists of two steps: firstly, from the appropriate percept clip to one intermediate clip (which serves to ‘label’ the given setup), and then onwards to an action clip representing a prediction. If the prediction is correct, then both of the connections traversed in the random walk will be strengthened in proportion to the agent’s reward.^6^ Once this process has been iterated often enough, the agent should have learned both (i) to label each of the percepts *s* with intermediate clips in $\mathcal {I}$, in the sense that the connections from *s* to one or several particular *i* are much stronger than to the others, and (ii) to choose correct outcome predictions for various experiments on the basis of those labels, in the sense that the connections from *i* to some actions are much stronger than they are to others.

Both of these sets of connections—from percepts to intermediate clips (representing assignments of labels to setups) and from intermediate clips to actions (encoding which labels are relevant to which experiments)—reflect patterns that the agent has learned in order to make sense of its environment. The present section provides a conceptual discussion of how certain properties and structures in the pattern of weights of these connections can be used to identify the abstract conceptual representations at play in the agent’s deliberations.

### Variable Identification Based on Connections from Percepts to Intermediate Clips

Before describing how we can identify the agent’s abstract representations of the environment’s hidden variables based on the weights of the connections in its ECM, it will be useful to specify more precisely what is meant by a ‘variable’ in this context. Formally, a variable can be characterised as an abstract property such that every setup instantiates one and only one value of that property. This definition is trivially satisfied by the hidden variables ‘mass’, ‘size’ and ‘charge’ in our running example. Importantly, this definition also implies that, for every variable, the set of values is jointly exhaustive and mutually exclusive with respect to setups, i.e., every setup maps to at least one value of a given variable, and no setup maps to more than one value of a given variable.

**Fig. 4:**
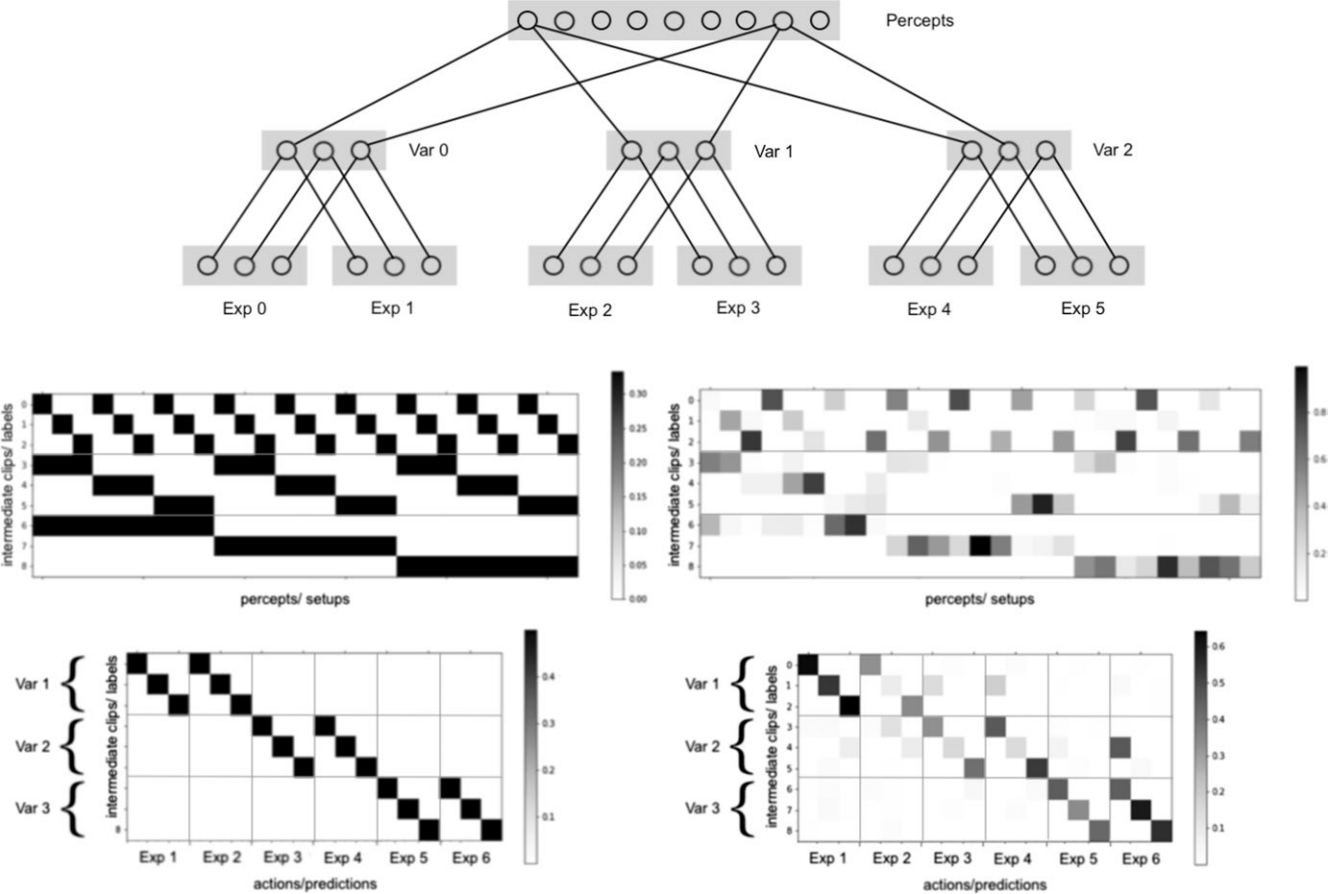
(Top) Structure of connections in the ideal ECM. For clarity, only connections from two percepts are shown, and all weak connections are suppressed. (bottom) Matrix representations of the ideal connections (left) and, for comparison, the corresponding connections formed by a real agent (right), showing separately connections from percepts to intermediate clips (top row) and from intermediate clips to experiment-predictions (bottom row). In order to showcase the characteristic structure of those connections, the intermediate clips are ordered according to the values they represent, as $V_0=0$, $V_0=1$, $V_0=2$, $V_1=0$, $V_1=1$, $V_1=2$, $V_2=0$, $V_2=1$, $V_2=2$. In the case of h-matrices learned by a real agent, the intermediate clips generally represent a random permutation of these values. However, they can be sorted in the same way by inferring the correct ordering based on an analysis of the connections, as detailed in Sect. 5

We can now detail what role such variables play in an agent’s deliberation on a learning task. Recall first that each intermediate clip can be interpreted as a label that the agent attaches to one or more percepts (specifically those that are strongly connected to the clip in question) and uses as a basis for predictions. The agent might develop labels that are only attached to a single percept, and are therefore best interpreted as meaning simply ‘This is setup *s*’. However, it is more efficient for the agent to label setups directly with the values of individual variables, since those are more naturally suited to making predictions about the outcomes of different experiments. To illustrate: the variable ‘size’ plays a role in the agent’s deliberations if and only if, in deciding which prediction to make for a given setup, they label the setup with a particular value for the ‘size’ variable (e.g., ‘big’, ‘small’, ‘medium’) and then choose the prediction on the basis of that label. This explication suggests that, when trying to identify the variables represented in the agent’s deliberative structures, we should expect each value of a variable to be represented by a label for setups, i.e., by an intermediate clip in $\mathcal {I}$. Accordingly, a whole variable should be represented by a *subset of intermediate clips*, denoted $\tilde{I}$, whose elements represent the various values of the given variable. Moreover, the sets representing different variables should be ‘mutually exclusive’ and ‘jointly exhaustive’ in the sense that for any setup *s*, the agent is disposed to label *s* by exactly one of the labels in the set. Roughly, this means that each percept connects ‘strongly’ to exactly one of the labels in the set, and ‘weakly’ to all the other labels in the set. If two setups $s_{1}$ and $s_{2}$ both link strongly to different labels in the set representing a variable, that means that the setups have different values for that variable. If they link to the same label, they are perceived as sharing the same value for the variable. The top half of Fig. 4 illustrates the kind of pattern in the ECM that allows us to identify representations of variables via the semantics described above.

In sum, then, the idea is this: in order to identify the abstract variables that are represented in the agent’s deliberative structures, we should attempt to identify the subsets of intermediate clips in the agent’s ECM that are mutually exclusive and jointly exhaustive with respect to setups. The functional role that these subsets play in the deliberations of a PS agent renders them susceptible to legitimate interpretation as internal representations of abstract variables.

Having specified a criterion for identifying the variables at play in our agents’ deliberations, we can now describe what it would look like for the agents to satisfy the central success criterion we put forward for the learning task described in Sect. 2.1: namely, that the agents form internal representations of the environment’s hidden variables, i.e., ‘mass’, ‘size’ and ‘charge’. Following the procedure described above, we would identify these representations with subsets of labels (intermediate clips in $\mathcal {I}$) that are jointly exhaustive and mutually exclusive with respect to setups. Specifically, we would expect each of the three variables to be represented by a separate (pairwise disjoint^7^) set of three intermediate clips (since each coarse-grained hidden variable has only three values) such that each of the 27 available setups is strongly connected to exactly one clip in each set. (One can verify that, for any clip in any set, there should therefore be 9 setups that map strongly to that clip, since for each value of charge/mass/size, there are 9 objects which have that value.) In the event that the agent forms a structure of this form in their ECM, we will be able to legitimately identify representations of the environment’s three hidden variables in their internal deliberative structures.

At this stage, it is worth pausing to reiterate a few important clarifications. Firstly, we stress that, in general (and in the specific example considered here), we assume that, for each hidden variable, there are several experiments whose outcomes are determined by the value that that variable takes for the given setup. If there were only one such experiment for a particular hidden variable, then the conceptual distinction between the hidden variable and the experiment that reveals it would be lost, and the intermediate clips would no longer represent abstractions, but would simply act as copies of the outcomes associated with the values of the hidden variables. The interpretation of intermediate clips as internal representations of the values of hidden variables is only principled and legitimate when the environment structure is rich enough to support abstraction, which in this case means that hidden variables are tested by multiple experiments.

Secondly, we stress that, while the outcome of an experiment M (‘What happens when I hold the test object next to this lump of metal?’, where the metal happens to be magnetised) can be predicted by knowing the value of a hidden variable $\mu$ (which an outside observer with knowledge on the subject might render as ‘Is it magnetic?’), the two are conceptually very different objects. Crucially, the experiment *M* is part of the agent’s repertoire of actions, whereas $\mu$ is a hidden variable, i.e., a property of the environment that is in principle inaccessible to the agent, and whose existence and role the agent can only *infer* from patterns in the way setups connect to (correct) predictions.

### Variable Identification Based on Connections from Labels to Experiment-Predictions

We turn now to presenting a second, alternative method of identifying the variables at play in the deliberations of our PS agents. We will see in Sect. 5 that the two methods produce largely identical results.

If, as above, one wants to group the intermediate clips/labels into subsets such that each set represents the different values of a single variable, one could also simply pick one experiment and map backwards to the labels that predict its various outcomes. The resulting set of labels is then naturally interpreted as representing the variable tested by the given experiment. Ideally, there should be exactly one such label for each prediction, since we have assumed that each of the predictions associated with a given experiment correspond to one specific value of the variable tested by that experiment. Moreover, if there exist experiments $e_1$, $e_2$ whose outcomes are predicted by the same variable, then one expects the sets of labels obtained in this manner to coincide. This allows one to verify that $e_1$ and $e_2$ are predicted by the same variable and, moreover, to identify which prediction of $e_1$ corresponds to the same value of the hidden variable as a particular prediction^8^ for $e_2$. On the other hand, if two experiments $e_1$ and $e_3$ are predicted by different variables, then one should expect that any label that is strongly connected to a prediction of $e_1$ is not strongly connected to any prediction pertaining to $e_3$. The expected pattern of connections is illustrated in the bottom half of Fig. 4.

In sum, the idea is that one can identify the different values of a single variable by identifying those labels that lead to all the different predictions of a single experiment. If there are two experiments whose various predictions are reached from the same set of labels, then these should be interpreted as being predicted by the same variable, whereas disjoint sets of labels herald experiments that reveal different variables.

Again, we can illustrate this second prospective semantics for identifying representations of hidden variables in the agent’s ECM by considering the example presented in Sect. 2.2. As before, the aim is that the agent form internal representations of the coarse-grained variables we interpret as ‘mass’, ‘size’ and ‘charge’. The new procedure for identifying these representations works as follows. For each experiment, we check whether there is a set of intermediate clips such that every clip in the set connects strongly to a different possible prediction for that experiment. For example, in the experiment in which the given object is placed on a scale, we check whether there exists a set $\tilde{I} \subseteq \mathcal {I}$ such that each $i \in \tilde{I}$ connects strongly to one of the three possible predictions for the experiment (‘high reading’, ‘low reading’, ‘medium reading’). If such a $\tilde{I}$ exists, then we can interpret $\tilde{I}$ as the agent’s internal representation of a variable that predicts the outcome of that experiment. Moreover, we expect it to be that (i) each of the clips in $\tilde{I}$ also connect strongly to exactly one prediction of the other mass experiment, and (ii) none of the clips in $\tilde{I}$ connect strongly to any of the predictions associated with any of the size or charge experiments. This fact allows the agent to deduce that there exists a single variable that predicts the outcomes of both the ‘scale’ and the ‘momentum’ experiment, but not the others. We, human scientists, might subsequently identify this variable as ‘mass’, but the essential inference that there exists such a variable can be made by the agent itself.

Finally, let us preempt a potential criticism that one might raise against this second procedure for identifying representations of variables in the agents’ ECM. Specifically, one might argue that by assuming that the agent knows that the number of values of each variable should correspond to the number of outcomes of some available experiment, we are essentially giving them a-priori knowledge about the hidden structure of their environment, and thereby trivialising the discovery task. However, we hold that, firstly, the agent can make the non-trivial inference that there exists an unobserved variable whose value predicts the outcomes of one or more experiments. Moreover, the agent learns to distinguish between several coarse-grained *intervals* of values that this variable can take that map to different predictions in the experiments. The semantics we are proposing makes no ontological claims about the values that the unobserved variable itself takes, but simply points out that there exist patterns in the environment that can be explained in terms of hidden variables. This is the essential insight that the agent distills, and it does not depend on any a-priori assumptions about the number of values this variable might take.

## Results

**Fig. 5:**
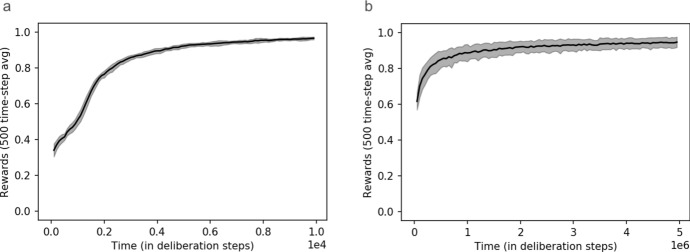
Comparison of the reward rate as a function of time achieved by **a** simple two-layer agents, which are incapable of abstraction, and **b** three-layer agents, which are capable of abstraction. Note the different time-scales required to achieve rewards around 90%: two-layer agents were trained for only $10^4$ rounds of interaction, while three-layer agents were given $T=5*10^6$. (Shaded areas denote standard deviation over an ensemble of 20 agents)

The first result of our simulations is that our three-layer agents learn to successfully predict the outcomes of setup-experiment pairs with success probabilities of at least $90\%$. One can compare how quickly the three-layer agent proposed here learns compared to a basic two-layer agent that simply tabulates the correct prediction for each percept-experiment pair. As shown in Fig. 5, two-layer agents learn much more quickly.

It should not come as a surprise that, in standard reinforcement learning tasks, three-layer agents are much slower when it comes to learning how to maximise rewards, since the extra clip-layer significantly complicates their deliberations and interferes with their memorisation of previous rewards. However, given that we are interested in engineering agents which are capable not just of maximising rewards, but also of forming interpretable conceptual representations of their environment, we do not take this to constitute a major problem. It is utopian to expect that one could satisfy the second of these success criteria without sacrificing something in the way of learning speed. But while they incur operational disadvantages regarding learning speed, we will see that three-layer agents accrue some major operational advantages pertaining to their ability to solve generalisation problems (see Sect. 5.3).

While establishing that agents learn how to make correct predictions and maximise rewards is important, the main point we want to make in this section is that agents with the three-layer ECM structure outlined above do indeed develop identifiable abstract representations of the environment’s hidden variables, i.e., they satisfy the central success criterion of the present work. To justify this claim, Sect. 5.1 details an analysis of the connections that the agent establishes between percepts and intermediate clips and how those represent abstractions and allow us to identify which subsets of intermediate clips represent variables, as outlined in Sect. 4.1. Analogously, Sect. 5.2 provides an analysis based on the connections from intermediate clips to actions (predictions), following Sect. 4.2. Finally, Sect. 5.3 turns to the problem of generalisation and demonstrates that, while two-layer agents are constitutionally incapable of solving the task (or of forming meaningful abstractions), our three-layer agents achieve a significantly better performance.

### Verifying Abstraction and Identifying Variables Based on Connections Between Percepts and Intermediate Clips

One way of analysing what the agent has learned is based on the conceptual considerations laid out in Sect. 4.1. We formalise the requirements of exhaustivity and exclusivity as follows:^9^ given a subset of intermediate clips $\tilde{I} \subseteq I$ that might represent (the set of values of) a hidden variable, we define functions $exh(\tilde{I})$ and $excl(\tilde{I})$ that assign to $\tilde{I}$ one real-valued indicator each, quantifying how well it satisfies exhaustivity and exclusivity, respectively. Intuitively, high values of $exh(\tilde{I})$ and $excl(\tilde{I})$ indicate that the elements of $\tilde{I}$ plausibly represent the values of a single variable identified by the agent.

Exhaustivity demands that each percept *s* be strongly connected to (at least) one clip in $\tilde{I}$. The condition is therefore violated, for a given *s*, if the probability of reaching any clip in $\tilde{I}$—technically, we take $\max _{i\in \tilde{I}} P(i|s)$—is much smaller than the probability of going to a clip outside the subset, which we quantify^10^ by $\max _{i\in I\backslash \tilde{I}} P(i|s)$. As a measure of exhaustivity, we take a (weighted, logarithmic) average of the ratio of these probabilities over all percepts,

3 $$\begin{aligned} exh(\tilde{I}) := \sum _{s} w_s \log \left( \frac{\max \limits _{i\in \tilde{I}} P(i|s) }{\max \limits _{i\in I\backslash \tilde{I}} P(i|s)} \right) , \end{aligned}$$

where $w_s$ is a vector of weights^11^. In an ideal agent and for subsets $\tilde{I}$ that actually represent a hidden variable, this measure is zero. Larger values can occur if the agent is more likely to go to clips inside $\tilde{I}$ than to any clips outside it, but, more importantly, values $<0$ herald a violation of exhaustivity.

Exclusivity demands that each percept *s* map strongly to no more than one intermediate clip in $\tilde{I}$. The condition is therefore violated, for a given *s*, if the second-largest probability of reaching a clip in $\tilde{I}$ is comparable to the largest one. As a measure of exclusivity, we take the (weighted, logarithmic) average of the ratio of these probabilities over all percepts,

4 $$\begin{aligned} excl(\tilde{I}) := \sum _{s} w_s \log \left( \frac{\max \limits _{i\in \tilde{I}} P(i|s) }{\text {sec}\max \limits _{i\in \tilde{I}} P(i|s)} \right) , \end{aligned}$$

with the same weights $w_s$ as above. In an ideal agent and for subsets $\tilde{I}$ that actually represent a hidden variable, this measure tends to plus infinity, whereas values close to 0 herald a violation of exclusivity. (The measure is non-negative by design.)

**Fig. 6:**
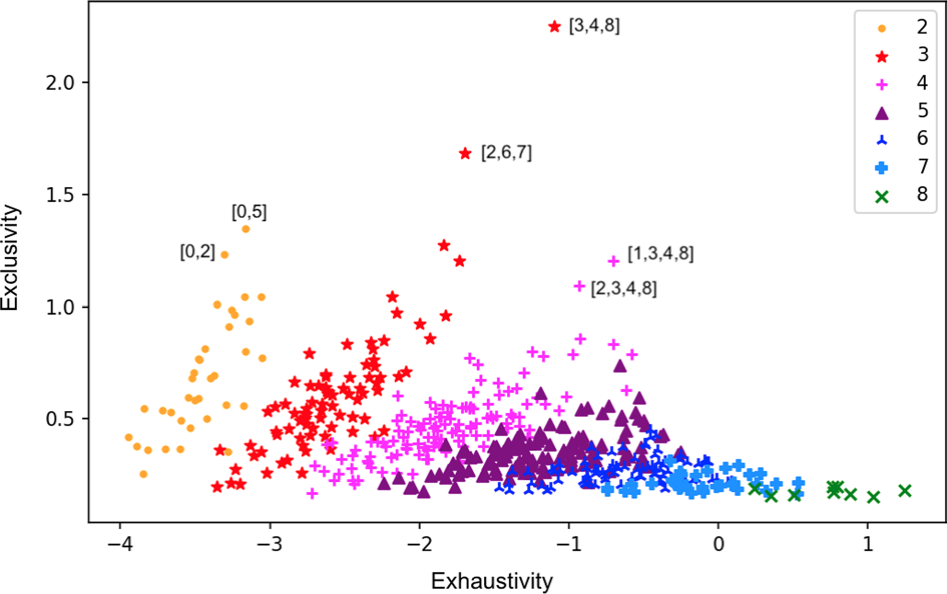
Measures of exclusivity and exhaustivity for all subsets of intermediate clips, for the agent specified in Sect. 3.2, with different symbols indicating the cardinality of the subset. Note how large subsets achieve comparatively high exhaustivity, but at the cost of violating exclusivity, whereas subsets of small cardinality have low exhaustivity but high exclusivity. Some of the subsets that achieve the highest values for both measures simultaneously are specified explicitly

Any subset $\tilde{I}$ that is close to representing (the values of) a hidden variable must have large values of both exhaustivity and exclusivity. To check which $\tilde{I}$ satisfy this condition, we plot the two measures for all subsets of the set of intermediate clips in Fig. 6. Based on this analysis, one can identify a few ‘good’ subsets; for example, in the particular agent analysed here, intermediate clips [3,4,8] are likely to represent one variable, while [2,6,7] are likely to represent another variable.

In addition to identifying particular subsets of intermediate clips, this analysis also reveals, for example, how many hidden intermediate clips are necessary to represent a hidden variable exhaustively (given by the cardinality of the ‘good’ subsets). For an ensemble of 20 agents in the standard setting described in Sect. 2.2, we obtain a value of $3.03\pm 0.19$, clearly revealing that the environment, in fact, contains hidden variables that take three distinct values each. A similar analysis can be performed based on the second layer of connections, as will be discussed in the following section. Figure 8 summarizes the results of this analysis and demonstrates how they allow one to read off essential parameters of the environment (in particular the number of values that the hidden variables can take) by tracking how the results change across different environments.

**Fig. 7:**
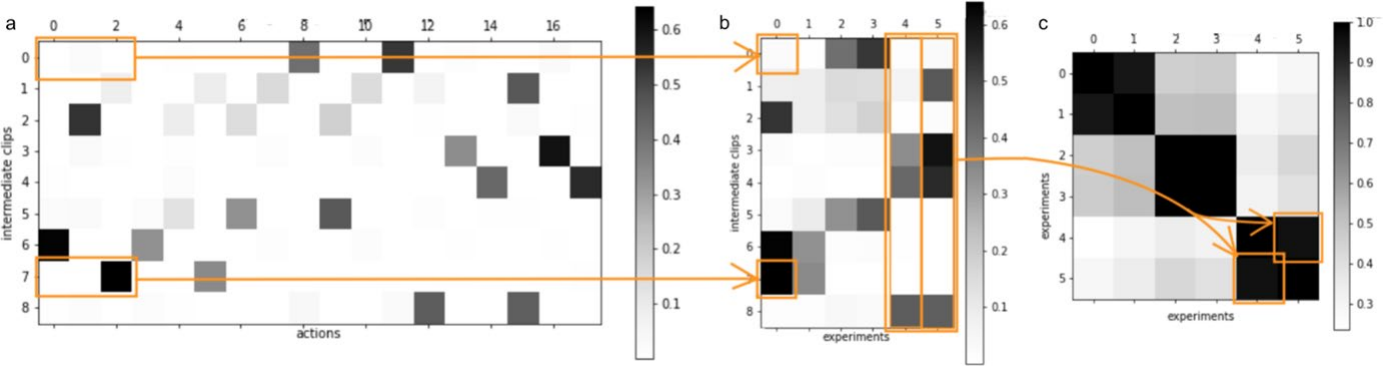
Identifying variables based on the connections from intermediate to action clips. **a** Transition probabilities from intermediate clips to predictions for each experiment *e*. These allow one to compute with how much certainty (quantified by the neg-entropy) each intermediate clip predicts the outcome of each experiment. **b** In a table of how well each intermediate clip predicts the outcomes of each experiment, comparing two columns (the ‘predictability profiles’) of two experiments allows one to judge how likely they are to involve the same variable. **c** The table of ‘predictability correlations’ between experiments has a striking block-diagonal structure, clearly showing that experiments 0 and 1 are predicted by one variable, 2 and 3 by another and 4 and 5 by a third. (Note that the suggestive ordering of pairs of correlated experiments in panel **c** is due to the way the environment was coded in our simulations. However, the high contrast of the correlation matrix allows one to identify related experiments in generic environments that do not have this ordering just as well. Note also that the correlation matrix is symmetric under transposition by construction, since the measure of correlation is independent of the order of the experiments being compared). Working backwards, one can identify in panel **b** that, for example, experiments 4 and 5 are predicted most prominently by intermediate clips [3,4,8], and one can further verify in panel **a** that those intermediate clips represent different values of the underlying variable, since they lead to mutually exclusive and jointly exhaustive predictions for the experiments in question

### Verifying Abstraction and Identifying Variables Based on Connections Between Intermediate and Action Clips

The procedure for analysing the connections from intermediate to action clips is summarised in Fig. 7. One begins by quantifying how strongly each intermediate clip predicts the outcomes of each experiment, which allows one to group experiments whose outcomes are predicted by the same subsets of intermediate clips together. Each such group is considered to stem from one hidden variable. Working backwards, one can then identify which intermediate clips represent values of each variable. This analysis reveals how many hidden variables are necessary to predict the outcomes of all experiments in question, and moreover how many—and, in fact, which—experiments are predicted by each of those variables. As for the intermediate clips, one can identify which intermediate clips represent the various values of each of those variables.

**Fig. 8:**
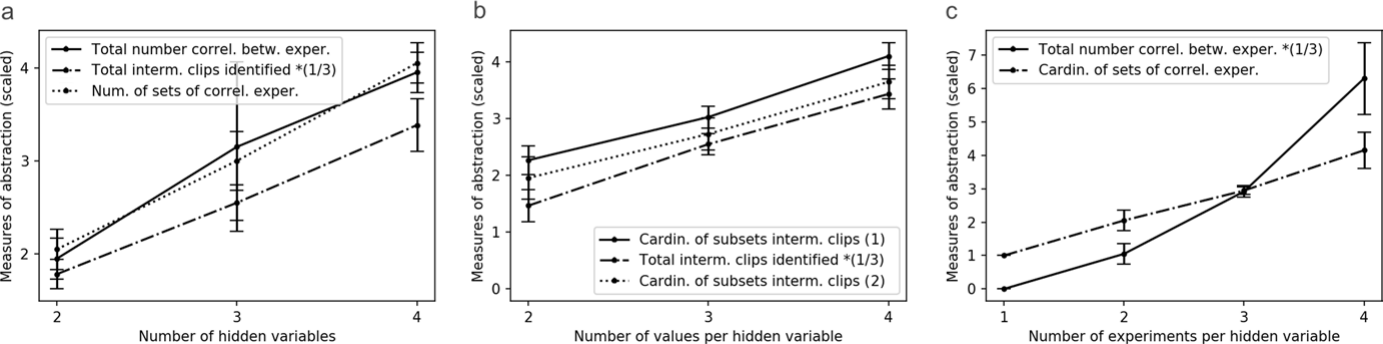
Identifying properties of the environment based on an analysis of the connections between percepts, intermediate clips and actions established by the agent (h-matrix): **a** One can read off the number of hidden variables $|\mathcal {V}|$ by determining the number of disjoint blocks in the correlation matrix depicted in Fig. 7c, or, indirectly, from the number of intermediate clips identified as representing values of variables (which should be $|\mathcal {V}|*|\mathcal {O}|$) or from the number of non-trivial correlations between experiments (which should be $|\mathcal {V}|*|\mathcal {E}|(|\mathcal {E}|-1)/2$). **b** One can read off the number of values each hidden variable can take from the cardinality of the sets of intermediate clips identified as representing single variables (based on either the connections to percepts (1) or the connections to actions (2)), or, indirectly, from the number of intermediate clips identified as representing values of variables (which should be $|\mathcal {V}|*|\mathcal {O}|$). **c** One can read off the number of experiments whose outcomes are predicted by each hidden variable by determining directly how many experiments are strongly correlated in the matrix depicted in Fig. 7c, or, indirectly, from the number of non-trivial correlations between experiments (which should be $|\mathcal {V}|*|\mathcal {E}|(|\mathcal {E}|-1)/2$). Note that agents in different environments were trained for different durations *T* (measured in interaction rounds), with the training times for each environment chosen such that the agents’ h-matrices settled into a clear pattern, as shown by the fact that the various measures used for analysing the abstractions formed by the agent no longer changed noticeably. Specifically, agents in the default scenario ($(|\mathcal {V}|,|\tilde{\mathcal {E}}|,|\mathcal {O}|)=(3,2,3)$) were trained for $T=5*10^6$ time-steps, whereas environments with different values used (**a**) ($|\mathcal {V}|=2$, $T=5*10^5$), ($|\mathcal {V}|=4$, $T=5*10^7$), ($|\mathcal {V}|=5$, $T=10^8$), (**b**) ($|\tilde{\mathcal {E}}|=1$, $T=5*10^5$), ($|\tilde{\mathcal {E}}|=3$, $T=5*10^6$), ($|\tilde{\mathcal {E}}|=4$, $T=5*10^6$), and (**c**) ($|\mathcal {O}|=2$, $T=5*10^5$), ($|\mathcal {O}|=4$, $T=10^7$). Error bars represent one standard deviation over an ensemble of 20 agents

For example, for the individual agent analysed here, the analysis identifies experiments 0 and 1 as being predicted by one variable, whose values are best represented by intermediate clips [2,6,7]; similarly experiments 2 and 3 are predicted by a hidden variable whose values are represented by intermediate clips [0,1,5], and experiments 4 and 5 are predicted by intermediate clips [3,4,8]. Let us compare this conclusion with the analysis based on the connections from percepts to intermediate clips, shown in Fig. 6: notably, while set [0,1,5] was not highlighted in Fig. 6, the two subsets that are identified most clearly in Fig. 6, [2,6,7] and [3,4,8], are the same ones found in the present analysis.

Regarding parameters of the environment, for an ensemble of 20 agents in the standard setting described in Sect. 2.2, we obtain the following measures:

- Number of hidden variables (distinct classes of correlated experiments): $3.00\pm 0.32$
- Number of experiments predicted by each variable (number of experiments with which each experiment correlates strongly): $2.05\pm 0.30$
- Number of values each variable can take (cardinality of the sets of intermediate variables identified): $2.73\pm 0.29$
- Number of distinct intermediate clips identified as best representatives of values of hidden variables: $7.65\pm 0.57$ (this should be num_features*num_values)

Figure 8 shows how these measures change across environments with different numbers of hidden variables, of values and of experiments per variable.

### Generalisation

**Fig. 9:**
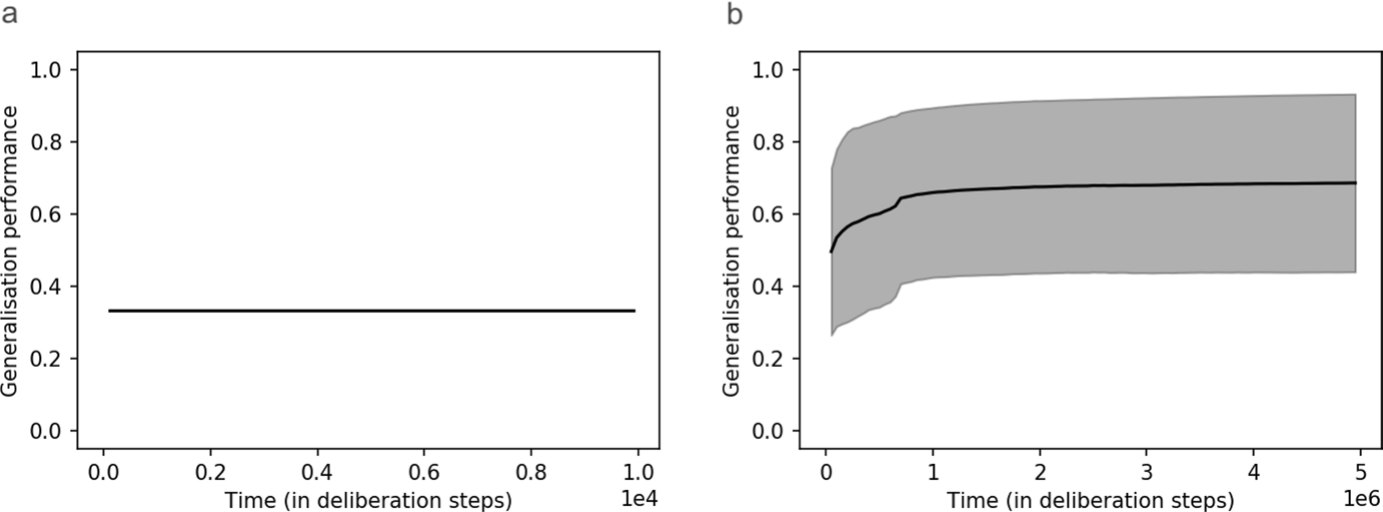
Performance on generalisation task over the course of training: **a** two-layer agents never go beyond chance level (1/number of possible outcomes, i.e., 1/3), whereas **b** three-layer agents achieve significantly higher success probability; in fact on par with their success rate at the percept-experiment pairs for which they did receive feedback. (Shaded areas denote one standard deviation over an ensemble of 20 agents)

To illustrate that the agent can, in fact, reap operational benefits from this construction, consider a problem where an agent only trains with a subset of objects and experiments, leaving out one object-experiment pair, but is then tested on the pair that it has never encountered. (In order to allow repeated testing at different stages of the learning process, these agents never receive feedback on the ‘test’ task.) Figure 9 illustrates how two-layer agents can only guess at random in that case, whereas an ensemble of our three-layer agents achieve significantly higher reward rates (on the validation test), of $(69\pm 25)\%$. This provides a concrete empirical vindication of the conjecture that the cognitive faculties of abstraction and generalisation are intimately related.

## Discussion

### On the Legitimacy of the Prospective ‘Concepts’

In Sects. 4 and 5, we presented an operational semantics for identifying the conceptual representations at play in the deliberations of PS agents, before empirically illustrating how these representations are formed and identified in concrete learning tasks. At this stage, one might be inclined to dispute the extent to which the identified representations are truly indicative of underlying conceptual thought. While we acknowledge that the defining characteristics of true conceptual thought are a topic of substantial ongoing philosophical debate and greatly exceed the ambit of the present work, it is worth highlighting that the semantics for concept identification presented here is consistent with (and supported by) some of the most influential philosophical theories of concept attribution.

Specifically, there is an influential view according to which the defining hallmark of conceptual thought is *compositionality*. On this view, an agent can only be said to truly possess a concept if they are able to use it in a completely general way that is not restricted to any particular cognitive context. For example, an agent could not truly be said to posses the concept ‘red’ if the only thought in which they are able to use the concept is ‘big red car’. In order to qualify as truly possessing the concept, the agent should be able to separate it from that one thought and use it to construct new thoughts like ‘small red van’ or ‘sparkly red necklace’. This idea was formally codified in Evans (1982) so called ‘generality constraint’, which posits that in order to qualify as engaging in genuine conceptual thought, an agent must be capable of entertaining all syntactically permissible combinations of the concepts they purportedly posses. So if the agent purportedly possesses the concepts ‘red’, ‘blue’, ‘heavy’, ‘van’, ‘house’ and ‘guitar’, they should be able to form the thoughts ‘red van’, ‘red house’, ‘red guitar’, ‘blue van’, ‘blue house’, ‘blue guitar’, ‘heavy van’, ‘heavy house’ and ‘heavy guitar’ (but not, e.g., ‘red blue’ or ‘house guitar’, which are not syntactically permissible). Variations of this generality constraint have been used to evaluate the prospective possession of genuine concepts by various animals. For example, Carruthers (2009) argues that the Australian digger wasp, which uses its body to measure the length of the sand towers it constructs, does not employ the concept of ‘length’ in a sufficiently compositional manner to warrant ascribing possession of the concept to it.

At this stage, it is pertinent to ask whether our ascription of concepts representing hidden variables to PS agents is consistent with Evans’ generality constraint or the requirement of compositionality more generally. Happily, it seems that the answer to this question is at least partially positive. To see this, recall that on our semantics, one of the central criteria for a set of labels to represent a variable is that they be jointly exhaustive with respect to setups. This means that for every object that the agent is capable of representing, they are strongly disposed to label that object with one of the values of the relevant variable. This in turn implies that each value of the identified variable will connect strongly to multiple objects. If we identify concepts with values of variables,^12^ then this means that the concepts formed by the agent are always applicable to multiple objects and therefore exhibit at least a moderate degree of compositionality. Although this is not enough to guarantee full accordance with Evans’ generality constraint, which demands complete and unrestricted compositionality, it is similar to the kind of partial compositionality that authors like Carruthers (2009) argue is sufficient for genuine conceptual thought.^13^

### From Abstraction to Generalisation

The majority of our discussion so far has focused on *abstraction*, i.e., the capacity to form abstract conceptual representations of salient features of one’s environment. A closely related (but importantly distinct) phenomenon is *generalisation*, meaning the capacity to utilise the knowledge that one has acquired through previous experiences to deal efficiently with *new* experiences that differ from everything one has previously encountered. It is natural to conjecture that an agent’s ability to solve generalisation problems is closely related to their ability to solve abstraction problems. One of the major payoffs of the present analysis is that we are able to provide a concrete empirical vindication of this conjecture, by showing that agents that form identifiable variable representations (in the sense described above) are better able to solve generalisation tasks than agents that lack the cognitive capacity to form these representations (see Sect. 5.3).

We also noted that this power comes at a cost, with simple two-layer agents reaching high rates of correct predictions much more quickly than the more sophisticated three-layer agents (see Fig. 5). In an environment where memorisation of percept-action pairs is a viable strategy, it may therefore be most efficient to employ a two-layer agent, which does not waste time looking for hidden variables. However, as one proceeds to larger, more complex environments, where the agent will more frequently encounter percepts it has not seen before, the ability to generalise (in particular by forming abstractions) becomes increasingly advantageous.

The task of facilitating meaningful generalisation in PS agents has been the focus of previous work, most notably by Melnikov et al. (2017). The generalisation capabilities exhibited by the agents considered in Sect. 5.3 go significantly beyond anything in this existing literature. Most importantly, our agents are able to successfully abstract and generalise in a way that does not rely on equipping them with a-priori knowledge regarding the structure of the environment. In contrast, the generalisation mechanisms described by Melnikov et al. rely on learning rules which implicitly encode a priori knowledge regarding the way in which the percept space can be coded by values of the environment’s hidden variables. In our framework, the agent discovers this hidden variable structure for themselves, and the very act of doing so facilitates their ability to generalise. No extra learning rule is required.

It is also instructive here to consider the relationship between abstraction and generalisation in the context of neural network architectures, which are of course remarkably successful in a wide array of practical generalisation problems that involve generalising the patterns encountered in the training set to deal with novel data in the test set. Typically, the networks are able to achieve this generalisation capability without developing any easily identifiable representations of abstract concepts. This suggests that, while abstraction can be a helpful basis for generalisation, as we have argued in the present work, it is not a necessary pre-requisite. On the other hand, the ability of artificial neural networks to generalise from their training examples to test instances has recently been cast into doubt, with the appearance of striking results of adversarial approaches: notably, Moosavi-Dezfooli et al. (2015) proposed an algorithm that systematically fools deep neural networks into misclassifying images by manipulating just a few pixels. Such results cast serious doubt on the reliability of broad and deep neural networks and highlight the importance of transparency in building more robust ML solutions.

### Transparency, Explanation and Abstraction

As well as allowing PS agents to accrue significant new operational capacities in generalisation tasks, the ability to form abstract conceptual representations also promises a number of other advantages. One of the most salient advantages relates to the problem of rendering the deliberations and decisions of PS agents fully communicable, explicable and transparent, a problem that becomes urgent whenever artificial intelligence is put to practical use in human society.

To see this, note first that the present work takes the first steps towards constructing an explicit symbolic interface through which PS agents can naturally articulate and communicate explanations of their reasoning processes and decisions. For example, once the semantics has been employed to identify the variables corresponding to ‘mass’, ‘size’ and ‘charge’ in the agent’s deliberations, it would be straightforward to implement an automatic explanation generator that provided explicit linguistic explanations of all the agent’s actions, e.g., ‘I predicted that the scale reading would be high because object 2 is heavy’. Although the exact definition of agent transparency is still a matter of significant controversy in the current literature (see, e.g., Chen et al., 2014; Lyons & Havig, 2014), it seems clear that the ability to automatically construct explicit explanations of an agent’s actions and deliberations constitutes a major step towards ‘transparency’ on all plausible interpretations of the term. For example, Chen et al. define agent transparency as ‘the quality of an interface (e.g., visual, linguistic) pertaining to its abilities to afford ... comprehension about an intelligent agent’s intent, performance, future plans, and reasoning process’. It is obvious that the present work makes significant strides towards bringing PS agents in line with this criterion.

### Prerequisites for Abstraction

Finally, one might wonder exactly what aspects of the PS agents described here are really responsible for their abstraction abilities. We have not yet provided any definitive argument showing that any particular aspect of the agents’ architecture is absolutely necessary for abstraction. However, it is clear that the present approach relied crucially on the introduction of the third intermediate clip layer. If there is any way to allow for similar abstraction capabilities in two layer agents, it will be radically different from the approach discussed here, which relies crucially on the additional structure from the third clip layer. So while we have not proved the necessity of a third clip layer for abstraction, we have at least identified an open problem regarding the possibility of abstraction in two layer agents. Furthermore, the fact that three layers of clips seems to be *sufficient* for some minimal degree of abstraction is itself an important realisation. There is undeniably much more work to do to untangle exactly what kinds of cognitive machinery are required for successful abstraction.

## Future Work and Conclusion

Finally, we conclude by highlighting promising avenues to be explored in future work.

The first avenue relates to one of the most distinctive and crucial cognitive capacities of human reasoners, namely the ability to identify and exploit correlations between variables in their environment. Here we have addressed one fundamental pre-requisite towards endowing PS agents with this ability by enabling them to identify variables that describe significant features of their environment. The next step is to construct a representation and learning rule that allows the agent to identify correlations between the different variables encoded in the ECM. We conjecture that doing so will allow us to further enhance the agents’ generalisation abilities. To see why, imagine that the agent is confronted with a setup *s* such that (i) they are already strongly disposed to label *s* with a value *v* of some variable *V* that is tested by an experiment *e*, and (ii) they are not strongly disposed to label *s* with any particular value of any variable $V^{*}$ that predicts an experiment $e^{*}$. In this case, the agent will already be good at predicting the outcome of experiment *e* when confronted with *s*, but they will not be able to reliably predict the outcome of $e^{*}$, perhaps because they haven’t yet had significant experience with the $e^{*}$/*s* pair. However, it may be that they have already noted a strong correlation between the variable *V* and some variable $V^{*}$ which they know is predictive of $e^{*}.$ In this case, it seems that they should be able to use their knowledge regarding the value of *V* that corresponds to *s* to guess a corresponding value for $V^{*}$, which would then allow them to make an educated guess regarding the outcome of $e^{*}$. In future work, we aim to develop a method for identifying correlations between an agent’s conceptual representations, and subsequently augment the PS learning dynamics in a way that utilises the observed correlations to allow for enhanced generalisation abilities.^14^

A second avenue for further work concerns the number of intermediate clips available to the agent. Throughout the present work, we have assumed this number to be fixed at a particular value. This is a significant assumption, which places a-priori restrictions on the kinds of abstractions that the agents are able to make. In future work, we intend to implement dynamics that allow the agent to autonomously alter its own architecture in a way that supports whatever kinds of abstraction are most useful for the learning task in which it is engaged. These dynamics would allow the agent to change the size of its label space over time as it gains information about the granularity and complexity of the hidden variables that characterise its environment. For example, one natural dynamic would be to ‘merge’ any two labels that look like duplicates of one another (in the sense that they define very similar probability distributions over action space). Another natural dynamic would be to ‘split in two’ any single label that is deemed to be too general and imprecise (in the sense that it defines an excessively flat probability distribution over action space). By implementing dynamics like these, we aim to make the concept formation scheme described here more autonomous, domain general and robust.

More generally, the present work takes the first steps towards allowing PS agents to autonomously develop symbolic interfaces through which they can articulate, refine and communicate their distinctive sub-symbolic reasoning dynamics. This opens up a host of new research avenues pertaining to the further development, integration and application of such interfaces.

## Footnotes

1 Again, the uniformity of the distribution over setups is a simplifying assumption that will be relaxed in future work, where we will also allow the experiments and hidden variables to stand in functional and probabilistic relationships to one another. We restrict ourselves to the simplest case here in order to focus on the central theme of conceptual abstraction without unnecessary technical distractions.

2 We stress that that ‘hidden variable’ terminology is not intended to reflect the usage of the terminology in the quantum foundations community. By ‘hidden variable’, we mean simply an environmental variable that is not directly observable for the agent.

3 Here we assume that whenever an experiment *e* tests a hidden variable *V*, each possible outcome of *e* (prediction for *e*) corresponds to exactly one possible value of *V*. It is possible to relax this assumption, but it plays a useful simplifying role in what follows.

4 This is closely related to the point that Block (1981) makes in his famous ‘Blockhead’ thought experiment, which is intended to refute the Behaviourist conception of intelligence enshrined in the Turing test.

5 For example, when looking at a traffic light an agent perceives a particular shade of green (determined by light and viewing conditions etc). They then disregard the particularities of that shade and simply label the experience as ‘green’, and then go on to choose an action (e.g., driving) on the basis of that label.

6 This is somewhat reminiscent of Hebbian learning, but note that in our model only transitions that led to a reward are strengthened.

7 We expect these sets of intermediate clips to be pairwise disjoint because knowing, for a given setup, that a variable $V_0$ takes a value $v_0$ or that a variable $V_1$ takes a value $v_1$ are two independent pieces of information, or labels, that can be assigned to the setup.

8 In general, it may be that $e_1$ and $e_2$ reveal different coarse-grainings of a single variable, so that some pair of values of that variable lead to the same prediction in $e_1$ but different predictions in $e_2$, while another pair of values is distinguished only by $e_1$ but not by $e_2$. In this case, one cannot identify labels and predictions for both experiments one-to-one. However, and more importantly, it still holds that a single *set* of labels connect strongly to all the predictions of $e_1$ and $e_2$, therefore still supporting the inference that these labels collectively represent a single variable that predicts the outcomes of both experiments. We have deliberately excluded such differently coarse-grained experiments in the scenario considered here in order to focus on the fundamental questions.

9 Note that the term ‘exhaustivity’ (and, analogously, ‘exclusivity’) can carry different meanings in the discussion of sets. One might say, given a set S and set S’ of subsets of S, that S’ is exhaustive if every element of S is also in some element of S’. However, we use the term with a different meaning, to describe a property of a single subset of intermediate clips with regard to their connections to percept clips, as detailed in the main text.

10 The case of the full set, $\tilde{I}=I$, has to be avoided, as this probability would then be zero.

11 For the data presented here, the weights $w_s$ for a given intermediate clip *i* are determined as follows: the percepts *s* are sorted according to the values *P*(*i*|*s*) and indexed with integers $ind_s=0,1,2,...S-1$ such that smaller *P*(*i*|*s*) carry larger indices. Then $w_s=(ind_s/(S-1))^3/N$, normalised to unit sum by choosing $N=\sum _{s}(ind_s/(S-1))^3$. This assigns higher weights (up to 1/*N*) to those percepts that have small transition probabilities, effectively highlighting possible violations of the exhaustivity condition.

12 So that the concept ‘red’ corresponds to one possible value of the colour variable, for example.

13 One worry that one might have about the approach described here concerns the problem of *empiricial underdetermination*—i.e., the fact that an agent’s conceptual and theoretical representations of the environment are often underdetermined by the empirical evidence, meaning that there are multiple different conceptual/theoretical frameworks that are consistent with observation. In simple toy examples like the one considered here, this problem is less pronounced, but it will be ubiquitous in more complex environments. We stress that we do not take our approach to contribute to any kind of a resolution of the problem of empirical underdetermination.

14 This is closely related to the more general problem of identifying PS agents’ subjective probability distributions over the set of identified variables. Doing this would open the door to the application of Bayesian inference methods in the context of PS, and with it, the possibility of realising Bayesian approaches to, e.g., causal inference (see, e.g., Spirtes et al., 2001), logical reasoning (see, e.g., Eva & Hartmann, 2018) and abduction (see, e.g., Douven, 1999) in PS agents.

## References

[CR1] Alvarez-Melis, D. & Jaakkola, T. S. (2018). Towards robust interpretability with self-explaining neural networks. In *Proceedings of the 32nd International Conference on Neural Information Processing Systems* (pp. 7786–7795). Curran Associates Inc.

[CR2] Barto AG, Baldassarre G, Mirolli M (2013). Intrinsic motivation and reinforcement learning. Intrinsically motivated learning in natural and artificial systems.

[CR3] Bengio Y, Courville A, Vincent P (2013). Representation learning: A review and new perspective. IEEE Transactions on Pattern Analysis and Machine Intelligence.

[CR4] Bermúdez JL (2003). Thinking without words.

[CR5] Biran O, Cotton C (2017). Explanation and justification in machine learning: A survey. In IJCAI-17 Workshop on Explainable AI (XAI).

[CR6] Block N (1981). Psychologism and behaviourism. Philosophical Review.

[CR7] Briegel HJ, de las Cuevas G (2012). Projective simulation for artificial intelligence. Scientific Reports.

[CR8] Carruthers P, Lurz RW (2009). Invertebrate concepts confront the generality constraint (and win). The Philosophy of Animal Minds.

[CR9] Chen B, Huang K, Raghupathi S, Chandratreya I, Du Q, Lipson H (2022). Automated discovery of fundamental variables hidden in experimental data. Nature Computational Science.

[CR10] Chen, J. Y. C., Procci, K., Boyce, M., Wright, J. L., Garcia, A., & Barnes, M. (2014). *Situation awareness-based agent transparency*. Army Research LaboratoryAberdeen Proving Ground: Technical report.

[CR11] Davidson D, Guttenplan SD (1975). Thought and talk. Mind and language.

[CR12] Douven I (1999). Inference to the best explanation made coherent. Philosophy of Science.

[CR13] Dreyse B (2011). Do honeybees have concepts?. Disputatio.

[CR14] Eva B, Hartmann S (2018). Bayesian argumentation and the value of logical validity. Psychological Review.

[CR15] Evans G (1982). The varieties of reference.

[CR16] Fahlman SE, Lebiere C (1990). The cascade-correlation learning architecture. Advances in Neural Information Processing Systems.

[CR17] Flamini F, Hamann A, Jerbi S, Trenkwalder LM, Nautrup HP, Briegel HJ (2020). Photonic architecture for reinforcement learning. New Journal of Physics.

[CR18] Hangl S, Dunjko V, Briegel HJ, Piater J (2020). Skill learning by autonomous robotic playing using active learning and exploratory behavior composition. Frontiers in Robotics and AI.

[CR19] Hangl, S., Ugur, E., Szedmak, S., & Piater, J. (2016). Robotic playing for hierarchical complex skill learning. In *2016 IEEE/RSJ International Conference on Intelligent Robots and Systems (IROS)* (pp. 2799–2804). IEEE.

[CR20] Higgins I, Matthey L, Pal A, Burgess C, Glorot X, Botvinick M, Mohamed S, Lerchner A (2017). beta-vae: Learning basic visual concepts with a constrained variational framework. ICLR.

[CR21] Higgins, I., Pal, A., Rusu, A., Matthey, L., Burgess, C., Pritzel, A., Botvinick, M., Blundell, C., & Lerchner, A. (2017b). Darla: Improving zero-shot transfer in reinforcement learning. In *Proceedings of the 34th International Conference on Machine Learning - Volume 70, ICML’17* (pp. 1480–1490). JMLR.org.

[CR22] Iten R, Metger T, Wilming H, del Rio L, Renner R (2020). Discovering physical concepts with neural networks. Physical Review Letters.

[CR23] Jerbi S, Trenkwalder LM, Poulsen Nautrup H, Briegel HJ, Dunjko V (2021). Quantum enhancements for deep reinforcement learning in large spaces. PRX Quantum.

[CR24] Lake BM, Salakhutdinov R, Tenenbaum JB (2015). Human-level concept learning through probabilistic program induction. Science.

[CR1000] Langley P, Simon HA, Bradshaw GL, Zytkow JM (1987). Scientific discovery: Computational explorations of the creative processes.

[CR25] Locatello, F., Bauer, S., Lucic, M., Rätsch, G., Gelly, S., Schölkopf, B., & Bachem, O. (2018). Challenging common assumptions in the unsupervised learning of disentangled representations. Retrieved from http://arxiv.org/abs/1811.12359.

[CR26] López-Incera A, Nouvian M, Ried K, Müller T, Briegel HJ (2021). Honeybee communication during collective defence is shaped by predation. BMC Biology.

[CR27] Lyons, J. B. & Havig, P. R. (2014). Transparency in a human-machine context: approaches for fostering shared awareness/intent. In *International Conference on Virtual, Augmented and Mixed Reality* (pp. 181–190). Springer.

[CR28] Mautner J, Makmal A, Manzano D, Tiersch M, Briegel HJ (2015). Projective simulation for classical learning agents: A comprehensive investigation. New Generation Computing.

[CR29] Melnikov AA, Makmal A, Briegel HJ (2018). Benchmarking projective simulation in navigation problems. IEEE Access.

[CR30] Melnikov AA, Makmal A, Dunjko V, Briegel HJ (2017). Projective simulation with generalization. Scientific Reports.

[CR31] Melnikov AA, Poulsen Nautrup H, Krenn M, Dunjko V, Tiersch M, Zeilinger A, Briegel HJ (2018). Active learning machine learns to create new quantum experiments. Proceedings of the National Academy of Sciences of the United States of America.

[CR32] Mnih V, Kavukcuoglu K, Silver D, Rusu AA, Veness J, Bellemare MG, Graves A, Riedmiller M, Fidjeland AK, Ostrovski G (2015). Human-level control through deep reinforcement learning. Nature.

[CR33] Mofrad AA, Yazidi A, Hammer HL, Arntzen E (2020). Equivalence projective simulation as a framework for modeling formation of stimulus equivalence classes. Neural Computation.

[CR34] Molnar, C. (2022). *Interpretable machine learning.* Independently published (2nd ed.). Retrieved from https://christophm.github.io/interpretable-ml-book/.

[CR35] Moosavi-Dezfooli, S., Fawzi, A., & Frossard, P. (2015). Deepfool: A simple and accurate method to fool deep neural networks. Retrieved from http://arxiv.org/abs/1511.04599.

[CR36] Nautrup HP, Delfosse N, Dunjko V, Briegel HJ, Friis N (2019). Optimizing quantum error correction codes with reinforcement learning. Quantum.

[CR37] Oudeyer P, Kaplan F, Hafner VV (2007). Intrinsic motivation systems for autonomous mental development. IEEE Transactions on Evolutionary Computation.

[CR38] Poupart P, Morales EF (2012). An introduction to fully and partially observable Markov decision processes. Decision theory models for applications in artificial intelligence: Concepts and solutions.

[CR39] Ried K, Müller T, Briegel HJ (2019). Modelling collective motion based on the principle of agency: General framework and the case of marching locusts. PLoS ONE.

[CR40] Samek, W., Wiegand, T., & Müller, K. (2017). Explainable artificial intelligence: Understanding, visualizing and interpreting deep learning models. Retrieved from http://arxiv.org/abs/1708.08296

[CR41] Sellam, T., Lin, K., Huang, I., Yang, M., Vondrick, C., & Wu, E. (2019). Deepbase: Deep inspection of neural networks. In *Proceedings of the 2019 International Conference on Management of Data, SIGMOD ’19* (pp. 1117–1134). New York: ACM.

[CR42] Spirtes P, Glymour C, Scheines R (2001). Causation, Prediction, and Search.

[CR43] Sutton RS, Barto AG (1998). Reinforcement learning: An introduction.

[CR44] Tiersch M, Ganahl EJ, Briegel HJ (2015). Adaptive quantum computation in changing environments using projective simulation. Scientific Reports.

[CR45] van Steenkiste S, Locatello F, Schmidhuber J, Bachem O (2019). Are disentangled representations helpful for abstract visual reasoning?. Advances in Neural Information Processing Systems.

[CR46] Wallnöfer J, Melnikov AA, Dür W, Briegel HJ (2020). Machine learning for long-distance quantum communication. PRX Quantum.

[CR47] Wiering M, van Otterlo M (2012). Reinforcement learning. State-of-the-art. Volume 12 of Adaptation, learning, and optimization.

